# The Flash-Lag, Fröhlich and Related Motion Illusions Are Natural Consequences of Discrete Sampling in the Visual System

**DOI:** 10.3389/fpsyg.2018.01227

**Published:** 2018-07-31

**Authors:** Keith A. Schneider

**Affiliations:** Department of Psychological and Brain Sciences, University of Delaware, Newark, DE, United States

**Keywords:** flash-lag effect, Fröhlich effect, motion illusions, discrete perception, temporal sampling

## Abstract

The Fröhlich effect and flash-lag effect, in which moving objects appear advanced along their trajectories compared to their actual positions, have defied a simple and consistent explanation. Here, I show that these illusions can be understood as a natural consequence of temporal compression in the human visual system. Discrete sampling at some stage of sensory perception has long been considered, and if it were true, it would necessarily lead to these illusions of motion. I show that the discrete perception hypothesis, with a single free parameter, the perceptual moment or sampling rate, can quantitatively explain all of the scenarios of the Fröhlich and flash-lag effect. I interpret discrete perception as the implementation of data compression in the brain, and our conscious perception as the reconstruction of the compressed input.

## Introduction

The human visual system makes consistent errors localizing the positions of moving objects (Linares et al., 2009). In the Fröhlich effect (Fröhlich, 1923; Kerzel, 2010), the perceived initial positions of abruptly appearing moving objects are displaced along their trajectories from their actual onsets, as are, in the flash-lag effect (MacKay, 1958; Nijhawan, 1994), their positions compared to actually aligned static objects. Attribute changes occurring within moving objects are perceived as, similarly, displaced (Zeki and Moutoussis, 1997; Eagleman and Sejnowski, 2007). These motion illusions have defied coherent explanation, but I demonstrate that they can be simply explained as artifacts of discrete temporal subsampling in the visual system (Stroud, 1954). The illusions are a natural consequence of a discrete perception hypothesis wherein visual input is broken into discrete perceptual moments, with moving objects registered only in their final positions during each moment. The single parameter model, with perceptual moments lasting 100–150 ms, quantitatively accounts for the measured phenomenology of the illusions. The possibility of discrete perception has long been contemplated (James, 1890, 1909; Bergson, 1911; Stroud, 1954). Bergson (1911) likened our stream of consciousness to a motion picture and contended that perception was necessarily discrete, with our thoughts and memories operating upon static images. James (1890) initially rejected the notion of discrete perception, writing, “Consciousness… does not appear to itself chopped up in bits" (p. 239) and held that only our understanding was discrete: “… we *take it in* in discrete pulses of recognition" (p. 622). Later, however, James (1909), influenced by Bergson, changed positions, writing, “Time itself comes in drops" (p. 232). I show that the discrete perception hypothesis elegantly explains a large class of visual illusions.

## Methods and Results

I hypothesize that visual input is sampled into discrete perceptual moments of duration *D*, and that any moving objects that might occupy a range of positions during the moment are registered at their final position occupied during each moment. The latter is a reasonable assumption, as motion deblurring techniques are thought to inhibit previous positions along a motion trajectory to prevent object persistence from confounding spatial extent (Burr, 1980), and the leading edge of a moving stimulus is most prominently evident in neural activity (Berry et al., 1999). We test this hypothesis by comparing its predictions to actual reports of visual perceptions of a variety of motion illusions.

**FIGURE 1:**
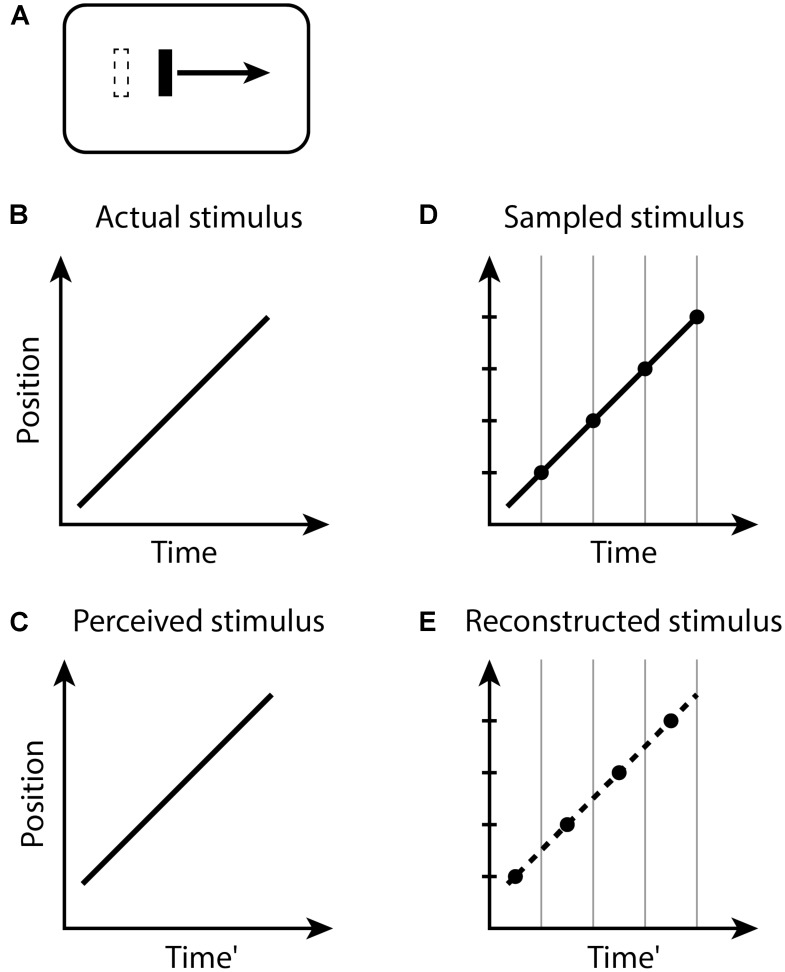
The Fröhlich effect. **(A)** A moving bar appears with an initial position indicated by the dashed outline, but the perceived initial position is displaced along the trajectory. Space–time diagrams **(B–E)**. **(B)** An object (black line) abruptly appears and begins moving. **(C)** The perception of this situation is that the object first appears in a position displaced along the trajectory of motion from the actual initial position. Note that the perceived Time’ axis is delayed by some amount relative to the actual Time in **(B)**. **(D)** The stimulus is sampled into discrete time windows, bounded by the vertical gray lines. The position of the moving object within each window is registered as the final position of the object in the window, indicated by the black dots. **(E)** The recorded positions of the moving object in time, indicated by the black dots, represent the position of the object at each time window. The smooth conscious perception of the moving object is reconstructed or interpolated between the discrete registered positions, as indicated by the dashed line.

In the Fröhlich effect (**Figure 1**), when the moving object appears, its first location is not registered until the end of the current perceptual moment. Because the perceptual moments are randomly positioned in time relative to stimuli, during a moment with duration *D*, the last position of the moving object will be displaced on average by a time *D/2* along its trajectory. The first observed position is therefore displaced from its actual position by the distance traveled by the object during this time, and the remaining trajectory of the object is reconstructed from this starting point. Typical estimates of *D*/2 for the Fröhlich effect are about 50 ms (Kerzel, 2010), yielding *D* ≈ 100 ms.

**FIGURE 2:**
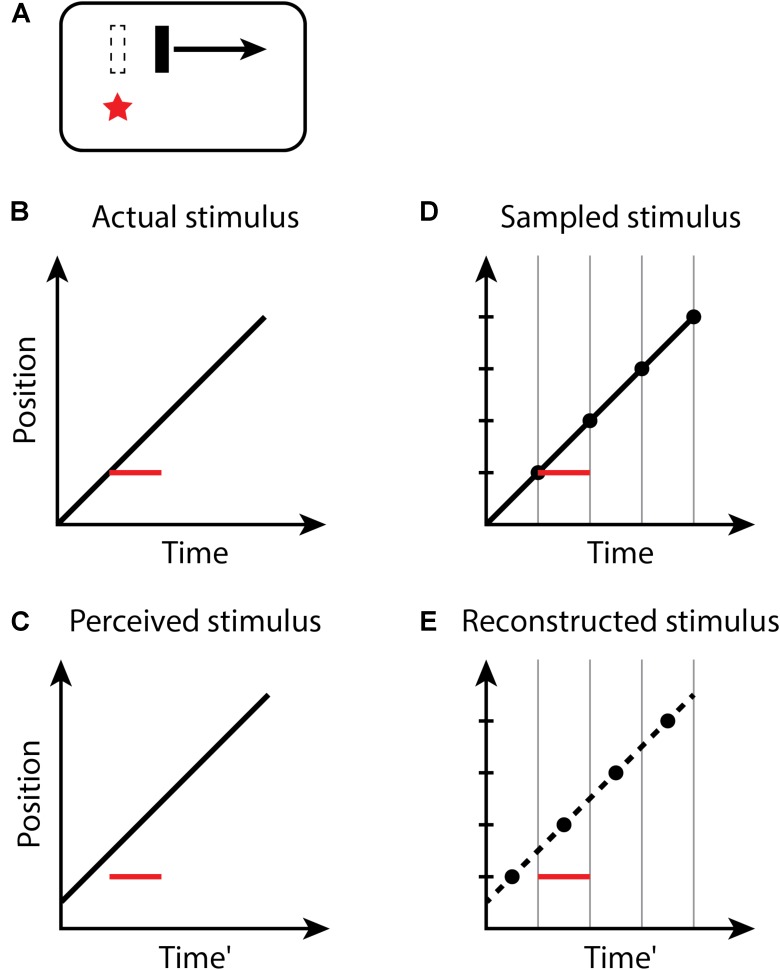
The flash-lag effect. **(A)** A flash (red star) occurs aligned with a moving bar (dashed outline), but the bar at the time of the flash is perceived as advanced along its trajectory. Space–time diagrams **(B–E)**. **(B)** An object (black line) is continuously moving through the visual field. At some time, a new static object (red line) flashes in alignment with the moving object. Note that the onset and duration of the flash within the moment do not affect its perception; the flash is thus depicted as filling the moment. **(C)** The perceived location of the flash appears to lag in behind the moving object. **(D,E)** as in **Figure 1**.

For the flash-lag illusion (**Figure 2**), the size of the effect observed on each trial of an experiment again is determined by the random temporal phase of the perceptual moment in relation to the stimuli. The size of the flash-lag should be uniformly distributed on the interval [0, *D*]. Both the flash and moving object are sampled in the same manner – the flash too is perceived at its final position within a moment, but its position is static, and its onset time and duration within the moment do not affect its perception. If the flash occurs at the last instant of the perceptual moment, and thus, the final position of the moving object within the moment is actually aligned with the flash onset, the flash-lag would be 0. If the flash onset occurs at the very beginning of a moment, the position of the moving object would be perceived at a time *D* later. The predicted mean magnitude of the flash-lag effect is *D*/2, which has been measured as 60.1−77.5 ms (Murakami, 2001). The discrete perception hypothesis makes consistent predictions for both the continual motion version of the flash-lag effect, above, for which the moving object continues to move along its trajectory both before and after the flash, as well as the flash initiated condition (Khurana and Nijhawan, 1995; Eagleman and Sejnowski, 2000b), in which the flashed object and the moving object both onset simultaneously. Note that the timing of the onsets of the flash and moving object is the same (Aschersleben and Müsseler, 1999; Eagleman and Sejnowski, 2000a; Arnold et al., 2009), as they appear within the same moment—only the position of the moving object is recorded at the end of the moment and is thus perceived as advanced from its initial position along its trajectory. For the flash terminated condition, in which the moving object halts at the same instant the flash appears, no misalignment between the flash and moving object is predicted, or observed experimentally (Eagleman and Sejnowski, 2000b; Rizk et al., 2009), since the last position of the moving object in any moment necessarily is aligned with the flash (because there is no motion after the flash).

An interesting case for the flash-lag effect occurs when the moving object abruptly reverses direction (Whitney and Murakami, 1998). Until time *D* before the reversal, there is no effect of the reversal on the perceived position or the moving object, which appears to lead the flash by time *D*/2 as usually. However, by time *D*/2 before the reversal, the average position of the moving object should be perceived as aligned with the flash, and by the time of the reversal, the average perceived position at the end of the moment would be *D*/2 back in the reversed direction. The average position should smoothly transition among these points, which is indeed observed experimentally (Whitney and Murakami, 1998; Brenner and Smeets, 2000; Whitney et al., 2000; Eagleman and Sejnowski, 2000b).

A variant of the flash-lag effect has been termed the flash-jump effect (Eagleman and Sejnowski, 2007), which is also similar to the color-motion asynchrony illusion (Zeki and Moutoussis, 1997). Here, the moving object abruptly changes one of its attributes, e.g., its color, during its motion trajectory, and the change is perceived, not in its veridical position, but rather displaced along the trajectory of motion. This is a natural consequence of the perceptual moments. The attribute change occurs during one perceptual moment, and the position of the moving object is registered in that moment as its last position within the moment. Therefore, the attribute change should be observed on average at a time *D*/2 past its actual time of occurrence.

**FIGURE 3:**
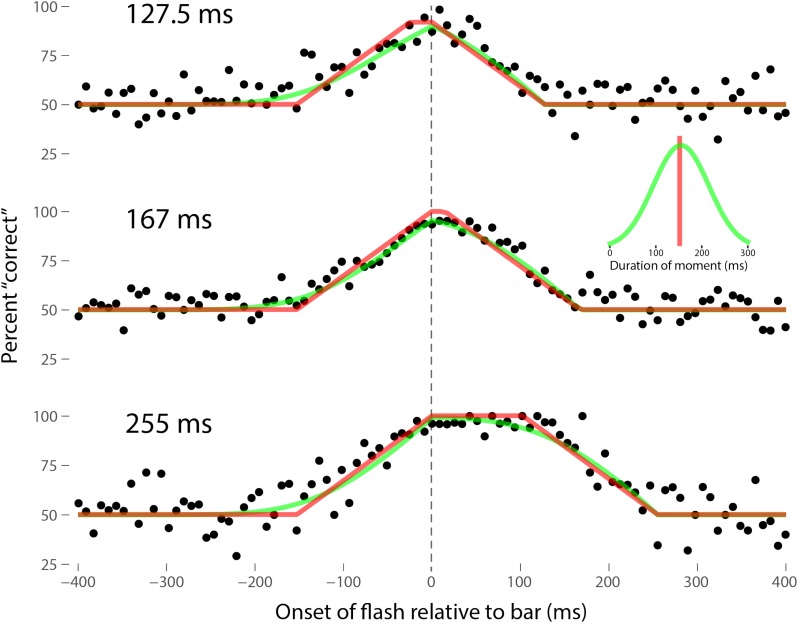
A fit of the discrete perception model to the random flash-lag data (Murakami, 2001). In this study, a bar was randomly displaced ±3° horizontally about fixation every 127.5 (top), 167 (middle), or 255 ms (bottom). An independent brief (8.5 ms) flash occurred every 3 ± 1 s, within ±50’ of fixation. The observers’ task was to report whether the flash occurred to the left or right of fixation. The data above were registered to the onset of the bar. The observers’ responses were scored as “correct” relative to this bar, as opposed to a subsequent or previous bar. The black points are averages from multiple trials for one subject (IM). The red lines are the fit of the single-parameter discrete perception model, linked across the three different bar durations, with *D* = 152.5 ms for this subject. The green lines indicated the best fit for *D* with a variable duration, distributed normally, as shown in the inset (*μ* = 154 ms, *σ* = 61 ms). The model shows the probability that this bar will be the one present at the end of the perceptual moment, averaged over all of the randomly placed possible phases of the moment.

**FIGURE 4:**
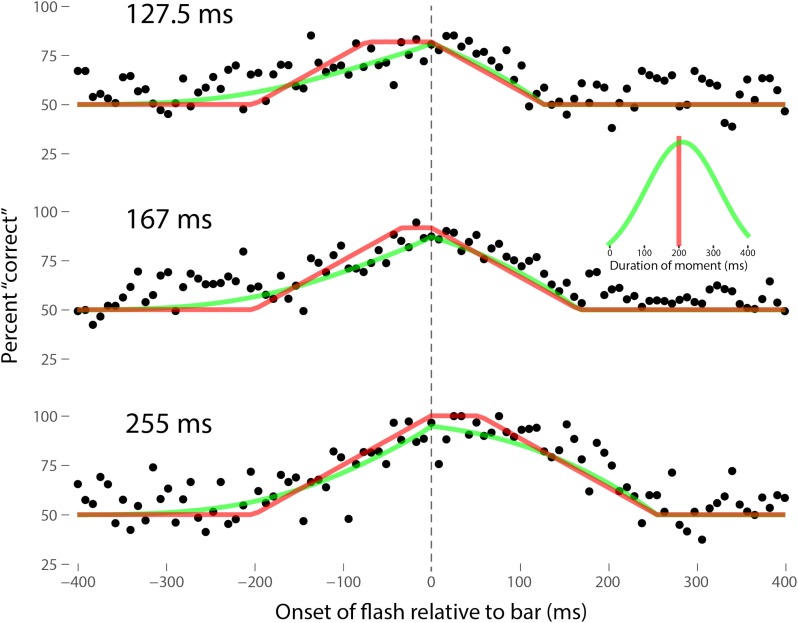
Data for the second subject, MM, as per **Figure 3**. Here *D* = 200.3 ms for the single-parameter model. For the two-parameter model with *D* normally distributed, *μ* = 212 ms and *σ* = 106 ms, as shown in the inset.

**FIGURE 5:**
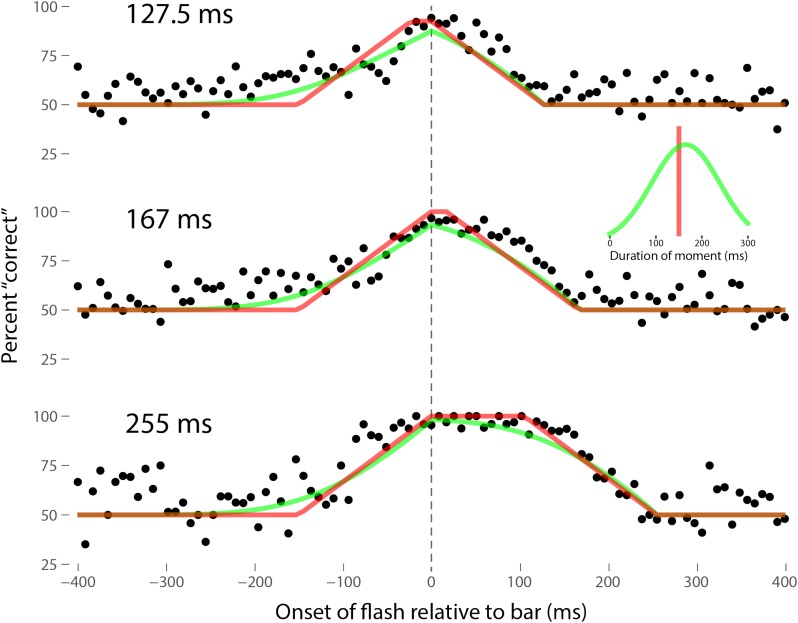
Data for the third subject, SN, as per **Figure 3**. Here *D* = 150.0 ms for the single-parameter model. For the two-parameter model with *D* normally distributed, *μ* = 165 ms and *σ* = 74 ms, as shown in the inset.

Perhaps the most comprehensive flash-lag data set was collected by Ikuya Murakami using random motion (Murakami, 2001), which probes the flash-lag effect on a fine time scale and intrinsically includes all of the motion reversal scenarios. Any model of the flash-lag effect needs to be able to explain the time course of these data, rather than just the mean magnitude of the effect that is typically reported in other studies. In this experiment, bars of fixed durations (127.5, 167, or 255 ms in separate experiments) were presented in random positions. Periodically, flashes (8.5 ms duration) were presented, and subjects had to indicate whether they perceived the flash to the left or right of each bar. Under the discrete perception hypothesis, the perceived position of the bar during each perceptual moment is its position at the end of the moment. Therefore, if the location of the bar when the flash occurs has not changed by the end of the moment, the subject will report the “correct” relative position. However, if the bar has moved to a different random position by the end of the moment, the subject’s judgment will be at chance. I sought to quantitatively fit the discrete perception model to this rich data set, which I obtained directly from Ikuya Murakami (**Figures 3–5** for the three separate subjects). To fit the data, we must determine the probability that the bar position at any instant in time is the same as the bar position at the end of the moment in which the flash occurred. Let Δ*F* be the onset time of the bar subtracted from the onset time of the flash (the *x*-axis in **Figures 3–5**), *t*_F_ the time of the flash, *t*_F_ - Δ*F* the onset time of the bar, *t*_F_ - Δ*F* -*B* the bar offset time, where *B* is the duration of the bar. Since the perceptual moment is randomly aligned in time relative to the flash, the end of the moment occurs at *t*_F_ + *D*φ, where *D* is the duration of the moment and φ ∈ [0,1] is the random phase. For a given Δ*F*, we need to determine the probability that the bar onset time occurs before the end of the moment, i.e., -Δ*F* < *D*φ, and that the bar offset occurs at or after the end of the moment, i.e., *B -* Δ*F* ≥*D*φ. *D* can be determined for each subject by sampling or integrating over φ and minimizing the error between the model and data. In principle, *D* could be drawn from any distribution, but the results show that this model, with a single uniform parameter *D*, linked among all of the three bar durations, adequately accounts for the data, yielding *D* = 152.5, 200.3, and 150.0 ms for the three subjects, respectively (the second subject with the largest *D*, had quite noisy data compared to the other two). For comparison, I also fit a two-parameter model, with *D* drawn from a normal distribution. **Figures 3–5** show the data for the three subjects, for the three separate bar durations. The red line is the fit for a uniform moment duration, and the green line is the best fit for *D* with a normally distributed duration (see insets). The discrete perception model accounts for the data simply and elegantly, whereas other models such as motion extrapolation and differential latency fail to fit the data or require modification (e.g., multiple differential latencies) (Murakami, 2001). Any model that involves a single timing or position differential cannot account for the ramps evident in the data and fits in **Figures 3–5**, which are fit by the sliding time window in the discrete perception model. This model needs to be tested on additional time-resolved data sets, in future experiments.

## Discussion

The leading explanations of these illusions are the differential latency (Whitney and Murakami, 1998) and postdiction (Eagleman and Sejnowski, 2000b) hypotheses. Both are approximations of the discrete perception hypothesis but do not completely explain all of the phenomenology. The differential latency hypothesis suggests that moving stimuli have a temporal processing advantage over unexpected or flashed stimuli and thus appear to lead them in time. The hypothesis is unable to explain the flash-initiated condition of the flash-lag effect, in which both the flash and moving object appear simultaneously, or the Fröhlich and the flash-jump effects, which have only one object and thus no possible latency differential. Moreover, temporal order judgments do not reveal temporal advantages for moving objects (Aschersleben and Müsseler, 1999; Eagleman and Sejnowski, 2000a; Arnold et al., 2009), nor is there any physiological evidence for such an advantage (Arnold et al., 2009).

The postdiction hypothesis (Eagleman and Sejnowski, 2000b) suggests that the position of a moving object is integrated for approximately 80 ms after a flashed object, to compare their positions. Similar to the discrete perception hypothesis, the postdiction hypothesis identifies the origins of motion illusions as spatial and not temporal, i.e., the position of the moving object is misconstrued at the time of the flash. It can explain the many variations of the flash-lag effect but has difficulty explaining the flash-jump and color-motion asynchrony illusions, because the flash itself is seen at a different location, and cannot account for the variability of the effects (Linares et al., 2009), which is naturally explained by the discrete perception hypothesis as the random phase alignment of the perceptual moments with the visual stimuli. It also seems unlikely that a flash would reset the integration period (Krekelberg and Lappe, 2000): triggering could work for isolated events, but it does not seem plausible for a continuous visual scene, although eye movements could be responsible for triggering (Meister, 1951). Triggered vs. independent models have been tested for temporal order judgments. Sternberg and Knoll (1973) proposed a triggered-moment model, in which the perceptual moment was triggered by a stimulus, and a perceptual-moment model, in which the timing of perceptual moments is independent of stimuli. Although, the predictions of the two models are similar, Schneider and Bavelier (2003) found that the perceptual-moment better explained data from temporal order and simultaneity judgments.

A revision of the postdiction theory (Eagleman and Sejnowski, 2007) suggests that moving objects are perceived to be biased in advanced positions along their trajectories— this is an accurate summary of the phenomenology, and the discrete perception hypothesis explains the underlying mechanism. Other studies have suggested temporal sampling (Brenner and Smeets, 2000) or positional averaging that is weighted toward the most recently sampled position (Roulston et al., 2006), explanations that are compatible with the discrete perception hypothesis. Another study claimed to be able to explain the flash-lag effect in terms of a bias to perceive certain velocities (Wojtach et al., 2008). They showed that the flash-lag effect was not linear with velocity, as other studies had showed, but rather was a logarithmic function, and they showed that this same function would result from the distribution of velocities that would be projected upon the retina due to the distribution of three dimensional motions in the natural world. However, this study does not provide an explanation for the flash-lag effect, but rather an explanation for biases in motion velocity; since the flash-lag effect is velocity dependent, it will reflect any biases in velocity perception.

One common observation, for both the Fröhlich effect and the variations of the flash-lag effect, is that attention can reduce the size of the effects (Müsseler and Aschersleben, 1998; Brenner and Smeets, 2000; Baldo and Namba, 2002; Namba and Baldo, 2004; López-Moliner and Linares, 2006; Sarich et al., 2007; Shioiri et al., 2010), though exceptions have been reported (Khurana et al., 2000). Since this occurs for suprathreshold stimuli, one should avoid explanations that involve changes in perceptual thresholds. Rather, I hypothesize that attention operates to enhance the available information by increasing the sampling rate (Sarich et al., 2007; Samaha et al., 2015; Samaha and Postle, 2015; Wutz et al., 2018), which need not occur uniformly throughout the visual field, thereby shortening the perceptual moment and diminishing the corresponding effect magnitudes. A similar variation in the duration of the perceptual moment was proposed for stimulus intensity (Shallice, 1964). The measured size of the perceptual moment does vary considerably among individuals and among different tasks.

Much of the spatiotemporal input to the visual system is redundant. Retinal circuits remove spatial redundancy, and this process continues in the cortex (Olshausen and Field, 1996), akin to compression techniques used to reduce the file size of digital images. The temporal mechanisms used by the brain are less clear (Nortmann et al., 2015), but temporal compression necessarily involves temporal subsampling. Note that low-pass filtering is theoretically equivalent to subsampling—a cubic spline for example can be exactly described by three discrete samples. Such discrete perception has long been debated (James, 1890, 1909; Bergson, 1911; Stroud, 1954), suggesting that the brain processes snapshots of the visual world similar to the frames of a motion picture, but has never been definitively proven. The hypothesis of discrete perception was perhaps most clearly articulated by Stroud (1954), and recently has been championed by VanRullen et al. (2010, 2014) and VanRullen and Koch (2003) to explain the continuous wagon wheel illusion (Purves et al., 1996). Stroud described “moments” of perception with a duration of approximately 100 ms, during which any events recorded by the visual system could not be temporally differentiated. However, his description did not precisely specify how events occurring during each moment were registered, making predictions from his theory difficult.

The discrete perception hypothesis described herein is agnostic as to the distribution of the frame rates—this is an empirical question. An examination in the distribution of subject responses could, for example, rule out a constant frame rate, but it would be more difficult to distinguish variable frame rate models from other perceptual hypotheses.

Our conscious perceptions of the world are fluid, not discrete; if we sample the world discretely, our continuous subjective perceptions must then be reconstructed from these samples. I propose that sampling/compression occurs during a feedforward processing cycle, terminating in object recognition, and that our conscious perceptions result from reconstruction/decompression during a feedback cycle. Since the reconstruction is based on sampled input, any temporal artifacts arising from the sampling process will be carried through to perception. Smoothing or interpolation between discrete frames would explain apparent motion as well as the phi phenomenon in which color changes of a moving object are perceived before they actually occur (Kolers and von Grünau, 1976). Interpolation could, similarly, explain the motion vectoring effects reported by Eagleman and Sejnowski (Eagleman and Sejnowski, 2007), in which moving objects or colors were observed in locations where they were not ever present. Perhaps the electrical alpha rhythms in the brain reflect the compression/decompression process, though their phase has at most small perceptual consequences (VanRullen et al., 2014), likely because the discrete perceptual moments completely tile the input space with no gaps among them. Previously, the alpha rhythms have been correlated with the magnitude of the flash-lag effect on each trial (Chakravarthi and Vanrullen, 2012), which is parsimonious with the present theory, and perhaps may provide a neural substrate for discrete sampling. Area MT is a likely candidate to perform a motion decompression function, as conscious perception is abolished if the feedback link from MT to V1 is disrupted (Pascual-Leone and Walsh, 2001), and if MT is damaged bilaterally, stroboscopic perception can result (Zihl et al., 1983), as if only the key frames in a compressed video were perceived.

## Conclusion

In 1869, Tolstoy (1869/2005):

For the human mind absolute continuity of motion is inconceivable. The laws behind any motion become comprehensible to man only when he breaks that motion down into arbitrary selected units and subjects these to examination. But at the same time this arbitrary sub-division of continuous motion into discontinuous units is the cause of much human error. (Vol. 3, Part 3, Ch. 1).

Such is the case in perception, where a discrete analysis of the continuous visual stream causes spatiotemporal misrepresentations that manifest as illusions of motion.

## Author Contributions

The author confirms being the sole contributor of this work.

## Conflict of Interest Statement

The author declares that the research was conducted in the absence of any commercial or financial relationships that could be construed as a potential conflict of interest. The reviewer SC and the handling editor declared their shared affiliation.
